# Fabrication and Compressive Properties of Low to Medium Porosity Closed-Cell Porous Aluminum Using PMMA Space Holder Technique

**DOI:** 10.3390/ma9040254

**Published:** 2016-03-30

**Authors:** Nur Ayuni Jamal, Ai Wen Tan, Farazila Yusof, Kondoh Katsuyoshi, Imai Hisashi, S. Singh, Hazleen Anuar

**Affiliations:** 1 Centre of Advanced Manufacturing and Materials Processing (AMMP), Faculty of Engineering, University Malaya, Kuala Lumpur 50603, Malaysia; ayuni_jamal@yahoo.com (N.A.J.); aiwen_2101@hotmail.com (A.W.T.); ramesh79@um.edu.my (S.S.); 2 Department of Mechanical Engineering, Faculty of Engineering, Kuala Lumpur 50603, Malaysia; 3 Joining and Welding Research Institute, Osaka University, 11-1 Mihogaoka, Ibaraki, Osaka 567-0047, Japan; kondoh@jwri.osaka-u.ac.jp (K.K.); imaihiro@niph.go.jp (I.H.); 4 Manufacturing and Materials Department (MME), Kulliyah of Engineering, International Islamic University Malaysia, P.O. Box 10, Kuala Lumpur 50728, Malaysia; hazleen@iium.edu.my

**Keywords:** porous Al, PMMA, space holder technique, closed pores, compressive behavior

## Abstract

In recent years, closed-cell porous Aluminum (Al) has drawn increasing attention, particularly in the applications requiring reduced weight and energy absorption capability such as in the automotive and aerospace industries. In the present work, porous Al with closed-cell structure was successfully fabricated by powder metallurgy technique using PMMA as a space holder. The effects of the amount of PMMA powder on the porosity, density, microstructure and compressive behaviors of the porous specimens were systematically evaluated. The results showed that closed-cell porous Al having different porosities (12%–32%) and densities (1.6478 g/cm^3^, 1.5125 g/cm^3^ and 1.305 g/cm^3^) could be produced by varying the amount of PMMA (20–30 wt %). Meanwhile, the compressive behavior results demonstrated that the plateau stress decreased and the energy absorption capacity increased with increasing amount of PMMA. However, the maximum energy absorption capacity was achieved in the closed-cell porous Al with the addition of 25 wt % PMMA. Therefore, fabrication of closed-cell porous Al using 25 wt % PMMA is considered as the optimal condition in the present study since the resultant closed-cell porous Al possessed good combinations of porosity, density and plateau stress, as well as energy absorption capacity.

## 1. Introduction

Porous Aluminum (Al) has attracted substantial attention due to its lightweight, low density and unique combination of physical and mechanical characteristics such as high strength-to-weight ratio, high stiffness, excellent impact energy absorption, high damping capacity, and good sound absorption properties [1,2,3,4,5]. Owing to its excellent properties, it is widely applied in circumstances where high strength and stiffness-to-weight ratio are concerned, as well as in the areas where energy absorption and permeability are appreciated [6]. For example, it has found increasing applications in various fields such as in the structural fields as heat exchanger, filters, flame retardant and silencers, as well as in the functional fields as crash energy absorption, sandwich panels and noise control [1,2,3,4]. Theoretically, porous Al can be classified into two groups, which are open-celled and closed-cell, depending on the connectivity of the cells [7]. Open-celled porous Al consists of interconnected pores whereas closed-cell porous Al is made of sealed pores that are surrounded by thin metallic cell walls [7].

Over the years, studies of open-celled porous Al in terms of its fabrication techniques, characterizations and mechanical performances have been extensively performed [8,9,10]. In contrast, there has been little investigation on the closed-cell porous Al, especially on that with low to medium porosity [11,12,13]. In recent years, closed-cell porous Al has drawn increasing attention, particularly in the applications requiring reduced weight and energy absorption capabilities such as in the automotive and aerospace industries [1,2,3,4]. Due to its ability to undergo large deformation with relatively constant stress, closed-cell porous Al with low to medium porosity level possesses higher moduli, strength and impact energy absorbing ability than its open-celled counterparts, as well as the closed-cell porous Al with higher porosity [3,4,13]. Although it has been eliciting much interest in many applications, literature on the production methods and mechanical performance of this closed-cell porous Al, particularly involving low to medium porosity, is still limited, and this is addressed in the current paper.

In general, closed-cell porous Al can be produced via two processing routes, known as liquid state processing (melt route) and solid state processing (powder metallurgy) [3,4,11,14]. Liquid state processing route is a direct foaming method starting from slurry of molten Al and gases are introduced into the melt through foaming agents or external gas sources to create bubbles, followed by subsequent solidification to produce a close-pored microstructure [3,4,15]. In contrast, the solid state processing route, often collectively called the space-holder method, is an indirect foaming method starting from mixing of metallic powder and foaming agents (also known as space holder), followed by compacting and sintering processes to obtain the closed-cell porous metal [11,12,14]. Although the liquid state processing route has been extensively practiced due to the simplicity of the processing, the as-produced porous metals are usually of low quality, characterized by non-uniform distribution of pore sizes and porosity. Moreover, additional material such as ceramic particle (typically silicon carbide (SiC) or alumina (Al_2_O_3_)) is generally added along with foaming agent to stabilize the molten Al, and thereby incurring additional processing cost [3,4,16].

To overcome the above-mentioned drawbacks, the more economical space holder method has been considered as a suitable method for the fabrication of closed-cell porous Al, and has attracted much research interest in recent years. By using this technique, closed-cell porous Al can be produced at a much lower temperature and under less severe chemical reactivity constraints with more precise control of process variables and pore size using an appropriate space holder material [17]. Titanium hydride (TiH_2_) and calcium carbonate (CaCO_3_) have been widely employed as the space holder materials for the successful fabrication of closed-cell porous Al by powder metallurgy route [1,2,18,19]. However, there are certain drawbacks in the use of these space holder materials. For example, economical limitation in terms of manufacturing cost arises when the expensive TiH_2_ is employed as the foaming agent and the decomposition of TiH_2_ leads to the formation of chemically inert hydrogen gas [18,19]. In contrast, although CaCO_3_ is an inexpensive alternative to TiH_2_, the decomposition temperature of CaCO_3_ is relatively higher (between 700 and 900 °C), which is significantly above the melting point of the Al [1,2]. One of the most important steps in the production of porous Al is the porous body stabilization. The high decomposition temperature of CaCO_3_ makes porous Al stabilization become more demanding and costly, thereby it is not cost effective. Therefore, further studies concerning the selection of a cost effective space holder material that can be well performed in the porous Al stabilization would be valuable.

Recently, polymethylmetacrylate (PMMA) has been proposed as one of the suitable space holder materials due to some outstanding characteristics such as excellent formability, good biocompatibility, and most importantly, it has a low decomposition temperature of around 360–400 °C, which causes almost no contamination on the resultant porous metal due to the ease in thorough decomposition. Moreover, it has a perfectly spherical shape, which is reported to have significant effects in affecting the mechanical properties of the resultant porous metal. Indeed, the successful fabrication of closed-cell porous metal by powder metallurgy route using PMMA as the space holder has been reported in some recent studies. Li *et al.* have successfully produced porous titanium with an average pore size of 200–400 µm and porosity in the range of 10%–65% by varying the amount and size of the PMMA particle [20]. In another study by Jeon *et al.*, they found that porous titanium with closed pore structure can only be obtained with 20 vol. % PMMA, whereas porous titanium with 70 vol. % PMMA showed the open cellular structure, suggesting that the pore structure of the resultant porous metal is dependent on the size and content of PMMA [21]. In a more recent study, Bi *et al.* fabricated porous magnesium with porosities between 1% and 40% by powder metallurgy using PMMA as the space holder and they found that the corresponding porosity of the resultant porous magnesium increased with increasing content of PMMA from 0 wt % to 30 wt % [22]. Although there has been progressive research in the fabrication of closed-cell porous metals using PMMA as the space holder, few efforts have been directed towards the fabrication of closed-cell porous Al with low to medium porosity. Therefore, this study serves to investigate the suitability of PMMA as the space holder material for closed-cell porous Al fabrication.

In the present study, the practical feasibility of PMMA powder as a suitable space holder material in the fabrication of closed-cell porous Al with low to medium porosity using powder metallurgy method was investigated. Specifically, the effects of PMMA content on the porosity, density, microstructure and compressive behavior of the porous Al were examined. We believe that this work will lay a good basis for future studies concerning fabrication of closed-cell porous Al with low to medium porosity via the powder metallurgy method.

## 2. Material and Methods

### 2.1. Raw Materials

All the materials unless otherwise stated were purchased from NovaScientific resources (M) Sdn Bhd (Selangor, Malaysia). Aluminum (Al) (99.9% purity, ~45 μm particle size), magnesium (Mg) (99.9% purity, ~10 μm particle size) and tin (Sn) (99.5% purity, ~45 μm particle size, Sigma Aldrich, Selangor, Malaysia) powders were used as the starting powders whereas polymethylmetacrylate (PMMA) micro-bead (99.9% purity, ~150 μm particle size) was served as the space holder material. In this study, 0.5 wt % of Mg powder and 1 wt. % of Sn powder were used as the sintering aids to assist Al in liquid phase sintering while crude oil of low sulfur content called CLE safe oil (JX Nippon Oil and Energy, Tokyo, Japan) was used to lessen powder mixture segregation in the mixing stage.

### 2.2. Preparation of Porous Al

**Figure 1:**
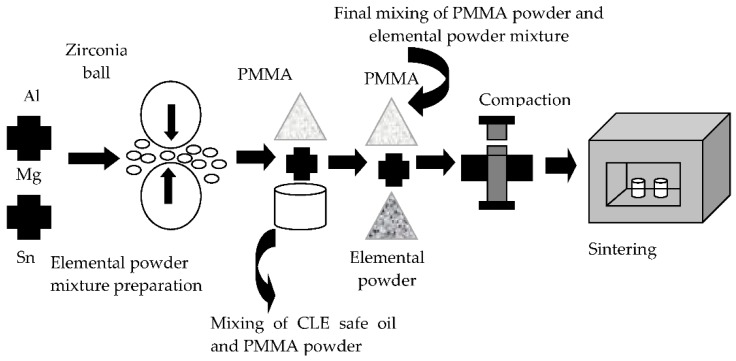
Schematic flow diagram of porous Al preparation.

Porous Al was prepared according to a simplified schematic flow diagram as shown in Figure 1, which consisted of mixing, compaction, sintering of compacted specimen and space holder removal stages. Firstly, the mixing of the elemental powder mixture consisted of Al, Mg and Sn was performed in a table-top ball mill with the powder-to-ball (Zirconia) ratio of 1:10 for 12 h. Prior to final mixing, a drop of CLE safe oil was added into the PMMA powder and mixed in a rotary miller for 1 h to promote the adhesion of elemental powder mixture on PMMA particles and to create uniform agglomerates. After that, the final mixing of the elemental powder mixture and the PMMA powder was carried out in a tubular shaker for another 1 h and the final powder mixture was then cold compacted in a cylindrical die of 10 mm in diameter and 12 mm in height at an applied pressure of 250 MPa. After cold compaction, the compacted specimen was first sintered at 450 °C for 1 h to remove the PMMA (space holder) content, followed by sintering of the specimen at 580 °C for 2 h under Argon ambient to obtain pure porous Al body. The sintered porous Al specimen was then washed with acetone and dried in an electrical oven at 90 °C overnight to remove impurities prior to characterization.

### 2.3. Evaluation of Density and Porosity of Porous Al

In this study, the amount of PMMA powder was varied between 20 wt % to 30 wt % to obtain porous Al of varying densities and porosities. The density of the sintered porous Al was determined by dividing the weight of the specimen over its volume. The porosity of the sintered porous Al was calculated using Archimedes principle by employing the following equation [23,24]:

Porosity, P = (*W*_ss_ − *W*_d_)/(*W*_ss_ − *W*_s_) × ρ_H2O_ (1)

where *W*_d_ is an unsaturated (dry) weight of porous specimen, *W*_ss_ is the saturated weight (assumed that all pores were filled with liquid) and *W*_s_ is the weight of saturated sample when submerged in liquid.

### 2.4. Microstructural Characterization

The morphology of the as-received starting powders, PMMA particles, the elemental powder mixture and the final powder mixture was observed using scanning electron microscope (SEM, Jeol JSM6500F, JEOL Ltd., Tokyo, Japan). For the microscopic examination of the sintered porous Al, the cross-section of the specimen was prepared. The microstructure and pore morphology of the specimen cross-section were then viewed by SEM.

### 2.5. Thermogravimetric (TGA) Analysis

The decomposition behavior of PMMA was performed using thermogravimetric (TGA, Perkin Elmer 7, PerkinElmer Inc., Waltham, MA, USA) analysis during heating up to 500 °C with 10 °C/min in Argon atmosphere.

### 2.6. X-ray Diffraction and Carbon Content Analysis

X-ray diffraction (XRD, PANanalytical empyrean 1032, PANalytical, Eindhoven, Netherlands) analysis was performed using CuKα radiation to identify the phase transformation of elemental powder mixture, final powder mixture and sintered porous Al. The XRD patterns were documented in the 2θ range of 20–80°. To ensure the purity of the sintered porous Al, carbon content analysis (LECO Co., CS-200, St. Joseph, MI, USA) was carried out to identify the remaining content of carbon (C) element of the powder mixture in every stage.

### 2.7. Compressive Behavior and Energy Absorption Capacity

The compressive strength of porous Al at different PMMA fractions was measured using universal testing machine (Shimadzu Autograph AGX 10 kN, Shimadzu Corporation, Kyoto, Japan) at room temperature with the crosshead speed of 1 mm/min and load cell of 10 kN. An average value of three samples was taken for the compressive response analysis. The energy absorption capacity, W, of the resultant porous Al was determined by the area under the stress-strain curve using the following Equation [25].

(2) $$W=\int_{0}^{\varepsilon }\sigma \;d\varepsilon$$

where σ and ε are the compression stress and strain, respectively. In this section, the relationship between relative density and energy absorption capability was emphasized and the following equation was implemented to calculate the relative density [3].

Relative Density = ρ^*^/ρ_s_ (3)

where ρ^*^ is the density of sintered porous Aland ρ_s_ is the theoretical density of Al which is equal to 2.7 g/cm^3^.

## 3. Results and Discussion

### 3.1. Morphology Characterization of Starting Powders

**Figure 2:**
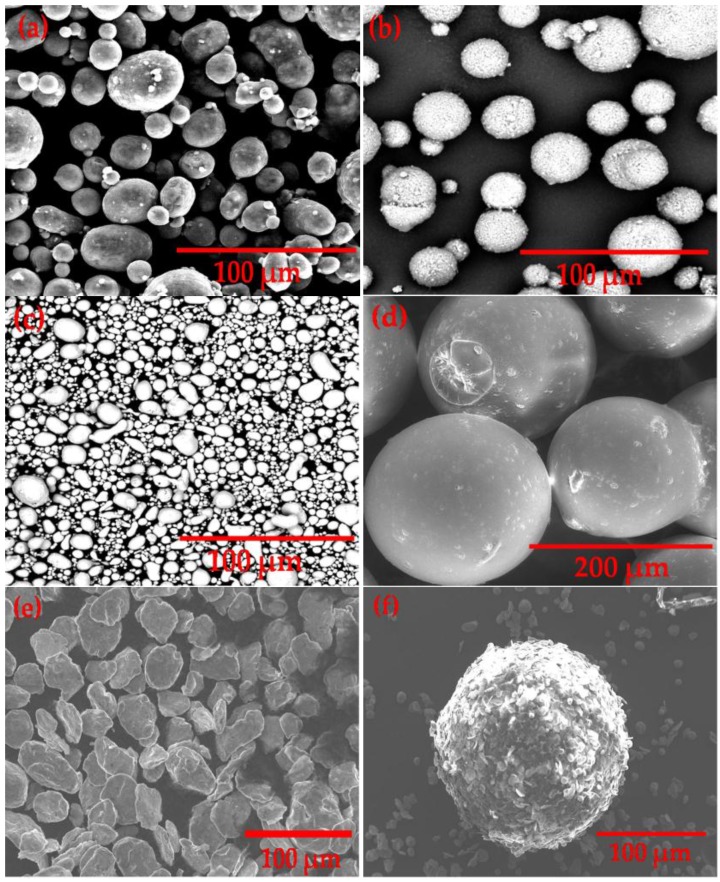
SEM micrographs of (**a**) Al powder; (**b**) Mg powder; (**c**) Sn powder; (**d**) PMMA particles; (**e**) elemental powder mixture after 12 h of mixing and (**f**) coating of elemental powders on the surface of space holder particle (PMMA).

Figure 2a–e shows the SEM images of each starting powder, elemental powder mixture and PMMA particle. Al and Mg powders were found to be mostly spherical in shape, with some of the Al particles in an irregular shape, as seen in Figure 2a,b, respectively. Similarly, perfectly spherical shapes were observed for the PMMA particle, whereas Sn powder was predominantly irregular in shape, as shown in Figure 2c,d, respectively. Since Al is a passive material that possesses a thermo-chemically stable Al_2_O_3_ film, it is crucial to disrupt or remove this film in order to form a strong metallurgical bonding between the Al particles. Therefore, in the present study, 1 wt % of Sn powder was added to increase the fluidity of Al during sintering whereas 0.5 wt % of Mg powder was served to enhance segregation of Sn particles on the Al surface [26,27,28]. As illustrated in Figure 2e, it is clear that 12 h of mixing time and 250 MPa of compaction pressure were sufficient to obtain a uniform distribution of elemental powder mixture without any powder agglomeration. The particles of elemental powder mixture were found to have lamellar structure with almost constant powder particle size and shape after the initial mixing stage. Such phenomenon can be attributed to the mechanical alloying process involving repeated welding, fracturing and re-welding of the powder particles that occurred during the mixing stage using the ball milling technique [29]. On the other hand, Figure 2f displays the successful coating of elemental powder mixture on the surface of PMMA particle, and the results showed that a homogenous powder distribution has been obtained through the use of CLE safe oil in promoting the adhesion of elemental powders on the PMMA surface [30].

### 3.2. Thermogravimetric (TGA) Analysis of PMMA

**Figure 3:**
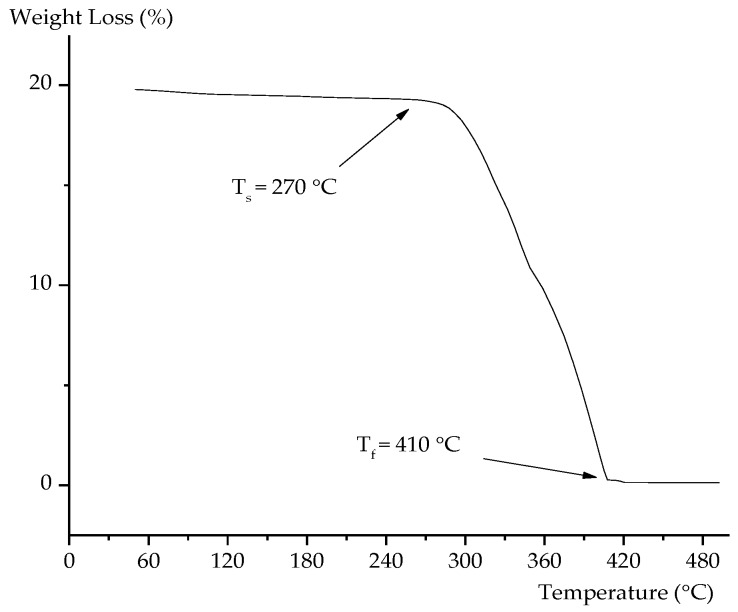
TGA curve of PMMA.

The TGA curve of PMMA powder is plotted in Figure 3, and the analysis showed that the thermal decomposition of PMMA spacer started at 270 °C (*T*_s_) and ended at 410 °C (*T*_f_). Therefore, it is believed that PMMA in the compacted Al can be completely removed in the sintering process during heating at 450 °C and then at 580 °C.

### 3.3. Microstructure Characterization of Porous Al

**Figure 4:**
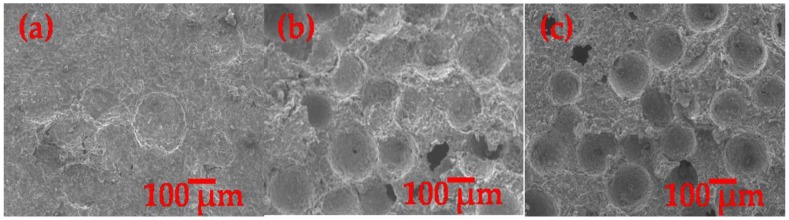
SEM micrographs of porous Al structure with (**a**) 20 wt % PMMA; (**b**) 25 wt % PMMA; (**c**) 30 wt % PMMA.

Figure 4a–c reveals the SEM micrographs of the resultant porous Al with 20 wt %, 25 wt % and 30 wt % of PMMA. It can obviously be seen that the pores in the resultant porous Al were mainly composed of closed macro-pore structure, especially in the case of 25 wt % and 30 wt % of PMMA. However, in comparison to Figure 4b,c, the appearance of pore formation was hardly seen on the porous Al with 20 wt % of PMMA (Figure 4a) due to the development of a dense cell wall, showing that 20 wt % of PMMA content was insufficient to create the desired closed pore structure in the porous Al. It is demonstrated that the macro-pores of the porous Al are obtained by the removal of the space holder particle and largely depend on the size and shape of the space holder particle [5]. As evident in Figure 4b,c, these macro-pores were found to be homogeneously distributed in the resultant porous Al and were isolated from each other by a distinct cell wall (average thickness of 51.11 μmin the case of 25 wt % PMMA and 45. 87 μm in the case of 30 wt % PMMA), further corroborating the formation of closed-cell structure in the resultant porous Al [31]. The morphology of these closed macro-pores were also observed to replicate the initial morphology of the spherical PMMA particle. The average pore size was around 159 μm, which is almost equal to the size of PMMA particle. Studies have shown that it is important to ensure that the pore formation resembles the morphology of space holder material in obtaining porous Al with better quality. Zhang *et al.* revealed a decline in compressive strength and elastic modulus of porous polymeric due to irregular pores formation prior to asymmetrical shape of space holder material [32]. Similar findings were also found in the study of Bekoz and Oktay, in which higher compressive properties of porous Al were obtained with the use of spherical spacer holder material as compared to the irregular one [33]. On the other hand, only a few micro pores were observed in the cell wall of porous Al as shown in Figure 4b,c and this is probably due to incomplete sintering of the Al particle. In the present study, the formation of the highest quantities of pores was obtained on the porous Al with the highest content of PMMA (30 wt %), followed by 25 wt % and 20 wt % of PMMA contents as seen in Figure 4a,c. From these findings, it is reasonable to infer that the fabrication of closed-cell porous Al using PMMA as the spacer is practically feasible, and porous Al with higher porosity can be obtained by increasing the weight percentage of PMMA particle.

### 3.4. Sintered Density and Porosity of the As-Produced Porous Al

**Figure 5:**
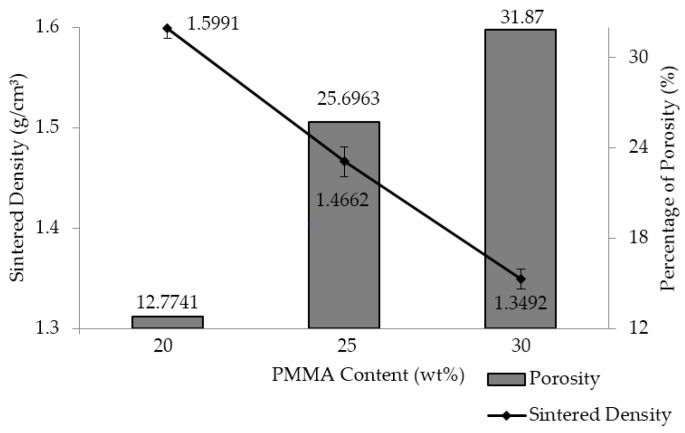
Sintered density and percentage of porosity as a function of PMMA content (wt %).

Figure 5 shows the sintered density and porosity of the as-produced closed-cell porous Al with different PMMA content. It can be observed that the sintered density of the porous Al decreased, and the porosity of the porous Al increased with increasing PMMA content. The sintered densities of the porous Al decreased from 1.6478 g/cm^3^ to 1.305 g/cm^3^ when the PMMA content was increased from 20 wt % to 30 wt %. In contrast, the porosity level of the porous Al increased from 12.77% to 31.87% as the content of PMMA was increased from 20 wt % to 30 wt %. Clearly, the addition of PMMA particle as the space holder material during fabrication reduced the density of the porous Al and consequently increased the porosity of the porous specimen. This can be attributed to the fact that a higher volume of closed pores is created when the PMMA content is higher. In this technique, the closed pores in the porous Al were created through the thermal decomposition of PMMA particles during sintering. Therefore, more closed pores were created as more content of PMMA was added, making the porous specimen possess higher porosity and become lighter. Similar observation was documented in the study of Manonukul *et al.* [11]. This observation is also in good agreement with our results obtained from the microstructural characterization as revealed in Figure 4a–c, in which a higher number of closed pores with thinner cell walls were found with increasing content of PMMA particles. It is therefore clear that the sintered density and porosity of the porous Al can be tailored by varying the content of the space holder material.

### 3.5. X-ray Diffraction Analysis

**Figure 6:**
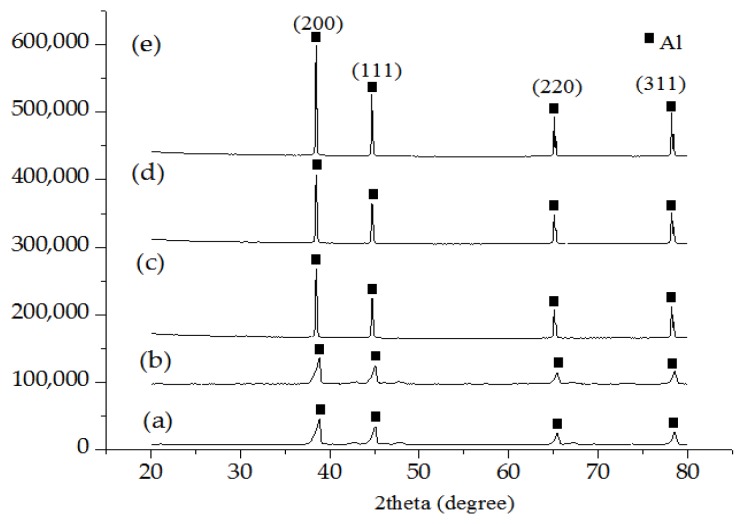
XRD patterns of (*a*) elemental powder mixture; (*b*) final powder mixture; (*c*) porous Al with 30 wt % PMMA content; (*d*) porous Al with 25 wt % PMMA content; and (*e*) porous Al with 20 wt % PMMA content.

The XRD patterns of elemental powder mixture, final powder mixture and sintered porous Al with different PMMA contents are revealed in Figure 6a–e. Considering the XRD diffraction patterns for final powder mixture with various PMMA contents are comparable, only one XRD diffraction pattern is chosen for discussion in this section. It is noted that Al rich phase was primarily found in the XRD pattern of all the samples, characterized by the (200), (111), (220) and (311) diffraction peaks at 38.87°, 45.42°, 67.16° and 78.54°, respectively. Moreover, no additional peaks were seen in the XRD patterns of the sintered porous Al, suggesting that Al did not react with PMMA spacer during sintering. As can be seen in Figure 6c–e, the peak intensities for all the sintered porous Al were found to be higher and sharper compared to the green compact specimen (final powder mixture), demonstrating the formation of crystalline Al during complete sintering. On the other hand, the presence of Mg and Sn peaks could hardly be detected, possibly due to the minor content of Mg and Sn powder employed (0.5 wt % and 1 wt %, respectively) during the fabrication process [34].

### 3.6. Carbon Content Analysis for Elemental Powder Mixture, Final Powder Mixture and Porous Al Specimen

It is known that methyl methacrylate (MMA, monomer) is the dominant volatile product of the decomposition of PMMA, and the decomposition of MMA was accompanied by formation of a number of low molecular weight stable species such as H_2_, CO, CO_2_, CH_4_, C_2_H_4_, CH_3_COOH in trace amounts [22]. To ensure that there is a complete removal of PMMA during the sintering process in producing pure porous Al, the chemical analysis of carbon (C) element before and after the sintering process was performed and the results are presented in Table 1. Initially, the carbon content of the final powder mixtures increased from 0.22 wt % to the range of 11.29 wt % to 13.03 wt % due to the addition of the PMMA into the elemental powder mixture. After sintering, the carbon content of the resultant porous Al was greatly reduced to the range of 3.87 wt % to 0.41 wt %, indicating that almost all the PMMA particles were completely decomposed during the sintering process, except for the resultant porous Al with 20 wt % PMMA [5]. The reason for this observation can be related to the entrapment of the PMMA particle in the Al matrix. It is postulated that most of the PMMA particles are well dispersed in the Al matrix and can be burnt off easily when there is sufficient amount of PMMA (25–30 wt % in this case) in the Al matrix, and hence only a small amount of carbon residue is left in the resultant porous Al after sintering. On the contrary, when the amount of the PMMA particles is insufficient (20 wt % in this case), some PMMA particles are completely enclosed by the Al matrix. These isolated PMMA particles are then unable to diffuse into the liquid part during sintering and thus become trapped in the Al matrix, resulting in higher amount of carbon residue in the resultant porous Al. Similar findings were also reported by Zhao *et al.* and Faizal *et al.* [35,36]. Therefore, it can be further concluded that the addition of 20 wt % of PMMA content was inadequate to produce closed pore structure in the fabrication of porous Al specimen via the space holder method.

### 3.7. Compressive Behavior and Energy Absorption Capacity of the Resultant Porous Al

**Figure 7:**
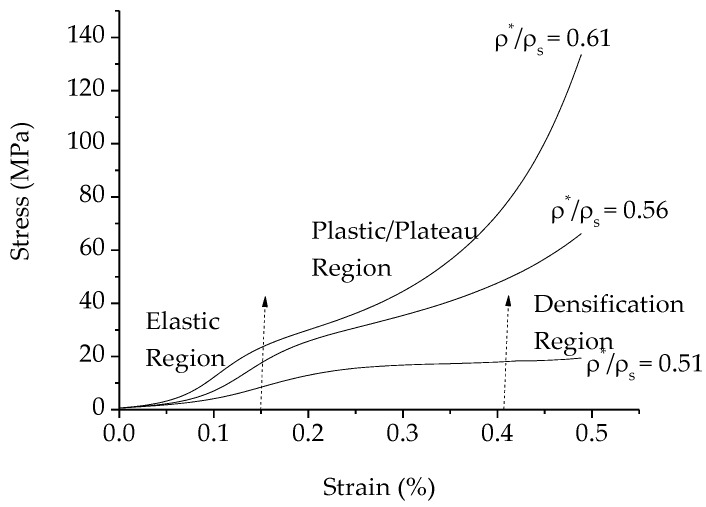
Stress-strain curves for different relative densities.

It has been reported that the most important microstructural feature that affects the compressive properties of porous Al is the relative density (ρ^*^/ρ_s_), which is the ratio of the density of the porous Al to that of the solid [3,4]. Therefore, the compressive stress-strain curves of the resultant porous Al having different relative densities are plotted in Figure 7, while the value of the relative density and the plateau stress are tabulated in Table 2. In this study, the plateau stress was taken as the average stress from 0.2% (yield point) to 50% strain [37]. It is observed that all curves exhibited the typical deformation pattern for closed-cell porous metals, which can be divided into three distinct stages: (1) a linear elastic region at the beginning of the deformation where cell wall bending and face stretching occur; (2) a plateau region that is characterized by a plastic deformation at a nearly constant flow stress and (3) a final densification region where the flow stress abruptly increased [3,16,20,38,39].

As illustrated in Figure 7, the plateau stress increased with increasing relative density. Due to the presence of the denser cell wall, higher buckling and bending deformation resistance of the cell walls are required during the elastic deformation, and thus porous Al with 20 wt % of PMMA content (relative density of 0.61) exhibited the highest plateau stress. In contrast, porous Al with 30 wt % of PMMA (relative density of 0.51) displayed the smallest plateau stress because elastic deformation may easily occur as a result of higher porosity of porous Al, resulting in reduction in the plateau stress. Similar findings were also reported previously [3,40].

During compression, a large amount of energy is absorbed during the stages of bending and collapse of the cell walls in the porous specimen, which occur mainly in the plateau region. Therefore, the plateau region plays an important role in determining the energy absorption capacity of the resultant porous Al. In the present study, the energy absorption capacity of the resultant porous Al with different relative densities was calculated from the area under the stress-strain curve according to Equation (2), and the results are presented in Table 2. It can be noticed that the energy absorption capacity of the porous Al increased with decreasing relative density, though this trend was not observed for the porous Al with lowest relative density (30 wt % of PMMA content). The highest value of energy absorption capacity of 3.65 MJ/m^3^ was achieved in the porous Al with medium relative density, which is porous Al with 25 wt % of PMMA contents. This is probably because the porosity and cell walls of this porous Al formed a more homogeneous pore structure than the other porous Al, and thus exhibited better compressive and energy absorption behavior. As can be seen in Figure 7, although the longest plateau length was observed for the porous Al with lowest relative density (30 wt % of PMMA contents), the energy absorption capacity was the lowest (as indicated by the small area under its stress-strain curve) as compared to the other counterparts, with its value comparable to that of the porous Al with highest relative density (20 wt % of PMMA contents). This phenomenon revealed that the cell structures in the resultant porous Al with 20 wt % and 30 wt % of PMMA particles could not support higher compressive loading before they fractured. The low value of energy absorption capacity in the porous Al with 20 wt % of PMMA contents is probably due to the inadequate pore formation as a result of insufficient amount of PMMA particles introduced. In the case of the porous Al with 30 wt % of PMMA contents, excessive space holder content caused the porous Al that possessed the highest porosity with thinner cell walls, and thus formed a weak porous Al structure that did not support further loading during the compression test. Taken together, it can be concluded that the optimum content of PMMA should be around 25 wt % since closed-cell porous Al with moderate plateau stress and highest energy absorption capacity could be produced.

## 4. Conclusions

The use of PMMA as a space holder material is practically feasible in successful fabrication of closed-cell porous Al with controlled porosity ranging between 12% and 32% by varying the amount of PMMA content in the range of 20–30 wt %. Our results demonstrated that the density of the porous Al decreased and the porosity of the porous Al increased with increasing PMMA content due to an increase in the density of closed-cell pores. The compressive stress-strain curves of the resultant porous Al showed that there was a decrease in the plateau stress and an increase in the energy absorption capacity with an increase in the amount of PMMA particles in general. However, the highest energy absorption capacity value was observed on the resultant porous Al with 25 wt % of PMMA content due to the porosity and cell walls of this porous Al forming a more homogeneous pore structure than the other counterparts, suggesting the optimum content of PMMA should be around 25 wt % under the present experimental conditions.

## Figures and Tables

**Table 1:** Carbon content of elemental powder mixture, final powder mixture and sintered porous Al with various PMMA contents. Data are presented in mean ± standard deviation.

Specimen	Carbon Content (wt %)
**Elemental powder mixture(Al-Mg-Sn)**	0.22 ± 0.52
**Final powder mixture with 20 wt % PMMA**	11.29 ± 0.52
**Final powder mixture with 25 wt % PMMA**	12.17 ± 0.43
**Final powder mixture with 30 wt % PMMA**	13.03 ± 0.61
**Sintered porous Al with 20 wt % PMMA**	3.87 ± 0.54
**Sintered porous Al with 25 wt % PMMA**	0.41 ± 0.15
**Sintered porous Al with 30 wt % PMMA**	0.43 ± 0.16

**Table 2:** Compressive behavior of porous Al with different PMMA contents. Data are presented in mean ± standard deviation.

PMMA Content (wt %)	Plateau Stress (MPa)	Relative Density (ρ* /ρ_s_)	Energy Absorption Capability (MJ/m^3^)
**20**	29.41 ± 0.42	0.61 ± 0.32	1.61 ± 0.60
**25**	24.76 ± 0.55	0.56 ± 0.28	3.65 ± 0.57
**30**	17.17 ± 0.49	0.51 ± 0.41	1.41 ± 0.44
